# Chitosan-Based Hydrogel in the Management of Dermal Infections: A Review

**DOI:** 10.3390/gels9070594

**Published:** 2023-07-24

**Authors:** Popat Mohite, Pudji Rahayu, Shubham Munde, Nitin Ade, Vijay R. Chidrawar, Sudarshan Singh, Titilope J. Jayeoye, Bhupendra G. Prajapati, Sankha Bhattacharya, Ravish J. Patel

**Affiliations:** 1 Department of Pharmaceutical Quality Assurance, A.E.T.’s St. John Institute of Pharmacy and Research, Palghar 401404, Maharashtra, India; mohitepb@gmail.com (P.M.); shubhamvmunde@gmail.com (S.M.); nitinade2@gmail.com (N.A.); 2 Department of Pharmacy of Tanjung Karang State Health Polytechnic, Soekarno-Hatta, Bandar Lampung 35145, Lampung, Indonesia; pudjirahayu@poltekkes-tjk.ac.id; 3 SVKM’s NMIMS School of Pharmacy and Technology Management, Jadcharla 509301, Telangana, India; vijay.chidrawar@gmail.com; 4 Department of Pharmaceutical Sciences, Faculty of Pharmacy, Chiang Mai University, Chiang Mai 50200, Thailand; 5 Department of Chemistry, Faculty of Science, Chulalongkorn University, Bangkok 10330, Thailand; titilope12@gmail.com; 6 Department of Pharmaceutics and Pharmaceutical Technology, Faculty of Pharmacy, Shree S. K. Patel College of Pharmaceutical Education and Research, Ganpat University, Mehsana 384012, Gujarat, India; 7 Department of Pharmaceutics, School of Pharmacy and Technology Management, SVKM’s NMIMS Deemed-to-be-University, Shirpur 425405, Maharashtra, India; sankhabhatt@gmail.com; 8 Ramanbhai Patel College of Pharmacy, Charotar University of Science and Technology, Anand 388421, Gujarat, India; ravishpatel.ph@charusat.ac.in

**Keywords:** hydrogels, chitosan, collagen, skin infections, crosslinking, polysaccharide

## Abstract

The main objective of this review is to provide a comprehensive overview of the current evidence regarding the use of chitosan-based hydrogels to manage skin infections. Chitosan, a naturally occurring polysaccharide derived from chitin, possesses inherent antimicrobial properties, making it a promising candidate for treating various dermal infections. This review follows a systematic approach to analyze relevant studies that have investigated the effectiveness of chitosan-based hydrogels in the context of dermal infections. By examining the available evidence, this review aims to evaluate these hydrogels’ overall efficacy, safety, and potential applications for managing dermal infections. This review’s primary focus is to gather and analyze data from different recent studies about chitosan-based hydrogels combating dermal infections; this includes assessing their ability to inhibit the growth of microorganisms and reduce infection-related symptoms. Furthermore, this review also considers the safety profile of chitosan-based hydrogels, examining any potential adverse effects associated with their use. This evaluation is crucial to ensure that these hydrogels can be safely utilized in the management of dermal infections without causing harm to patients. The review aims to provide healthcare professionals and researchers with a comprehensive understanding of the current evidence regarding the use of chitosan-based hydrogels for dermal infection management. The findings from this review can contribute to informed decision-making and the development of potential treatment strategies in this field.

## 1. Introduction

The existence and growth of pathogens such as viruses, bacteria, parasites, or fungi within the host organism cause infectious diseases to arise or return repeatedly every year. These pathogens could spread among individuals. The specific clinical symptoms are intimately correlated with the type and extent of the microbial agents’ harm because they frequently emit toxins that disrupt the regular operation of organs and systems [1,2]. Skin infections are widely prevalent infectious diseases and are recognized as the fourth leading cause of human illnesses [3]. Skin problems are a broad category of dangerous, challenging-to-treat illnesses brought on by bacteria, fungi, and viruses. The impact of skin infections on an individual’s health, psychological well-being, daily functioning, and social engagement is substantial. In 2013, it was projected that the socioeconomic burden of skin disorders in the United States totaled USD 11 billion in indirect expenditures and USD 75 billion in direct healthcare costs [4]. The incidence of skin conditions increases proportionally with an aging population. As individuals grow older, the natural elasticity and flexibility of the skin decline, resulting in more frequent tears in its integrity. These openings allow pathogens to invade the dermis, leading to skin infections. However, to fight against infections and stop the onset of serious illnesses, the skin’s microbiota and the antimicrobial substances produced various layers of defense system [5]. As pathogens continue to evolve and mutate, and as the population ages, our inherent immune defense system becomes increasingly compromised. Consequently, it becomes challenging for the immune system to mount a timely and effective response against the actions of these pathogens.

Transdermal patches and various semisolid dosage forms, including hydrogels, are widely recognized delivery strategies for addressing dermal conditions. Hydrogels have been extensively investigated and reported in scientific research. Three-dimensional network architectures are developed by these hydrogels’ hydrophilic polymer chain composition that absorb and hold enormous quantities of liquid or physiological fluids [6]. Moreover, the properties and performance of hydrogels are significantly affected by the structure of their molecules [7]. Crosslinking agents and polymer chains are the two main constituents of hydrogels [8]. There are both natural and artificial polymer chains in hydrogels [9]. Natural polymers commonly used in hydrogel formulations include agarose, alginate, chitosan, collagen, gelatin, hyaluronic acid, and cellulose derivatives [10]. These polymers possess inherent biocompatibility and biodegradability, making them suitable for biomedical applications. Tailoring the chemical and physical attributes of artificial polymers, including polyethylene glycol, polyvinyl alcohol, poly-N-isopropyl acrylamide, and polyacrylic acid, enables the development of hydrogels with specific features [11]. Crosslinking agents are introduced to connect the polymer chains and form the network structure of the hydrogel. These agents can be either physical or chemical. Changes in pH, temperature, or additional environmental variables can cause physical crosslinking, including reversible interactions, such as hydrogen bonding or physical entanglement. Chemical crosslinking involves covalent bonding between polymer chains, which is typically achieved through chemical reactions, such as free radical polymerization or condensation. Common chemical crosslinkers include glutaraldehyde, ethylene glycol dimethacrylate, and di-isocyanates. The molecular structure of hydrogels can be further modified by incorporating functional groups or additives to enhance specific properties. For example, introducing reactive functional groups allows the conjugation of bioactive molecules, such as peptides or growth factors, to promote cell adhesion and tissue regeneration [12]. Additionally, incorporating nanoparticles or micelles within the hydrogel matrix can provide controlled drug release, enhanced mechanical strength, or stimuli-responsive behavior [13,14]. These structural parameters can be tailored to meet the specific requirements of various applications, including tissue engineering, drug delivery, biosensing, and wound healing [15]. Hydrogels have gained significant attention in the field of wound healing and the treatment of skin infections. Their unique properties enable them to be suitable for managing various types of skin infections. Therefore, the present review elaborates on utilizing chitosan as a gel-forming excipient in managing various dermal infections.

## 2. Skin: The First Line of Defense

**Figure 1:**
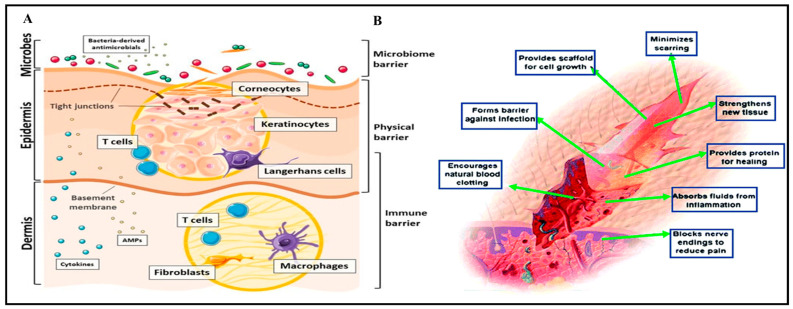
Division of the skin and its mechanisms of protection against microbial penetration. Reproduced with permission under CC BY 4.0 license [24] (**A**). Hydrogel made of chitosan, used to treat wounds, generates a three-dimensional tissue network and activates macrophages to fight tumors. Reproduced with permission under CC BY 4.0 license [25] (**B**).

The skin protects against the loss of internal substances and external stimuli, including microbial invasion, as the largest and most vulnerable organ in the human body (Figure 1). The epidermis, which is the outermost layer; the dermis, which is located beneath the epidermis and primarily made up of connective tissues; and the hypodermis, which is deeper subcutaneous tissue made up of connective and adipose tissues, make up the three main layers that make up the skin [5,16,17]. The body’s largest organ, the skin, acts as the body’s primary defense against external dangers and as the first line of defense. The epidermis, dermis, and hypodermis comprise its three principal layers [18]. The outermost layer, the epidermis, acts as a protective shield. It consists of multiple layers of cells, with the outermost layer constantly shedding dead skin cells.

Specialized cells called keratinocytes within the epidermis produce keratin protein, which provide strength and protection [19]. The dermis is a thicker layer that lies beneath the epidermis and is home to various organs, including blood vessels, hair follicles, sweat glands, sebaceous glands, and sensory receptors. The dermis supports the skin structurally and harbors immune cells that contribute to pathogen defense. The fat cells that comprise the hypodermis or subcutaneous tissue, the layer with the most excellent depth, provide insulation, cushioning, and energy storage [20]. The skin is an effective barrier against pathogens, chemicals, and physical injuries. It prevents the entry of microorganisms and harmful substances into the body. The epidermis, with its tightly packed cells and the presence of antimicrobial peptides, forms a physical and chemical barrier. Additionally, the skin’s slightly acidic pH inhibits the growth of certain microorganisms [21].

The skin’s immune system plays a crucial role in its defense function. Langerhans cells in the epidermis detect foreign substances and initiate immune responses. Other immune cells, including T cells and macrophages, are also present in the skin, aiding in eliminating pathogens [22]. In summary, the skin’s anatomy, with its distinct layers, works harmoniously to provide a robust first line of defense. This unique system protects the body from external threats, preserving its integrity and well-being [23].

## 3. Types of Skin Infections (Bacterial, Fungal, and Parasitic)

Skin infections can be classified into three main types based on the causative agents: bacterial, fungal, and parasitic. Here is an overview of each type:

***Bacterial infections*:** Bacterial skin infections are caused by various bacteria that invade and multiply within the skin. Common bacterial infections include (1) impetigo: characterized by red sores that rupture and form yellowish crusts; (2) cellulitis: a deeper infection that affects the skin and underlying tissues, causing redness, swelling, and warmth; and (3) folliculitis: inflammation of hair follicles, resulting in small red bumps or pustules.

***Fungal infections*:** Fungal skin infections are caused by different types of fungi that thrive in warm, moist environments. Common fungal infections include athlete’s foot (Tinea pedis): affecting the feet and causing itching, redness, and cracking of the skin; ringworm (Tinea corporis): characterized by circular, red, scaly patches on the skin; jock itch (Tinea cruris): occurs in the groin area, resulting in itching, redness, and a rash; and Candidiasis: caused by the Candida fungus, typically affects areas with skin folds, such as the armpits and groin.

***Parasitic infections*:** Parasitic skin infections are caused by parasites that infest and feed on the skin. Common parasitic infections include scabies: caused by tiny mites that burrow into the skin, leading to intense itching and a pimple-like rash; pediculosis (lice infestation): an infestation of lice on the scalp, body, or pubic area, causing itching and the presence of lice or their eggs; and cutaneous larva migrant occurs when the larvae of certain parasites penetrate the skin, resulting in itchy, winding tracks.

### 3.1. Molecular Structure of Hydrogels

**Figure 2:**
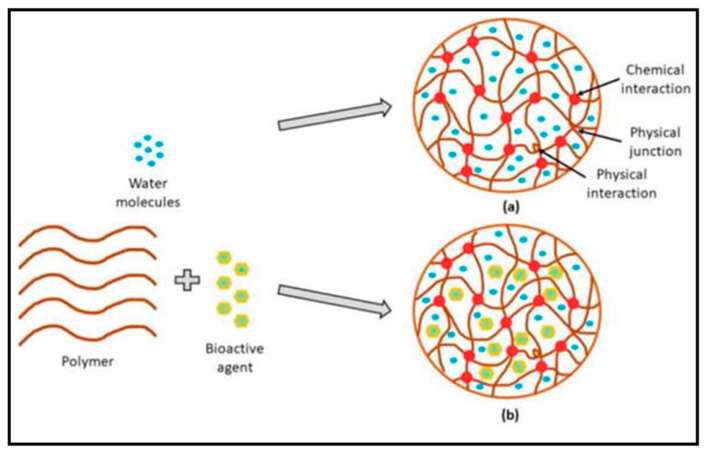
Hydrogels’ molecular composition without bioactive (**a**) and fortified with bioactive (**b**). Reproduced with permission from [35] under CC BY 3.0.

Hydrogels are a three-dimensional (3D) crosslinked network of polymer chains that absorb enormous amounts of fluid due to their hydrophilic functional groups (hydroxyl, carboxyl, amide, and amino), which adhere to the polymeric backbone [26]. The term “hydrogel” was invented for the first time in 1894 by van Bemmelen. The molecular structure of hydrogels is functionally characterized by the mesh size that is crosslinked physically through hydrogen bonding and chemically via covalent bonding (Figure 2). The hydrogel’s primary feature is its capacity to absorb large amounts of water when water molecules disperse into the structure of the hydrogel.

Hydrogels are polymeric networks that can undergo degradation over time, and crosslinking is crucial for their structure formation and degradation control. Crosslinking involves the formation of stable connections between polymer chains, resulting in the formation of a 3D network structure. Synthetic and natural polymers can fabricate hydrogels that have 3D structures that are able to hold large quantities of water or biologic fluids. The choice of the polymer depends on the desired properties and applications of the hydrogel, which can offer precise control over the hydrogel’s properties and can be modified to enhance biocompatibility and biofunctionality [27]. Moreover, synthetic hydrogels are often preferred when specific mechanical or chemical properties are required for a particular application [28]. Chitosan and gelatin are frequently utilized in hydrogel compositions [29]. These natural polymers offer inherent biocompatibility and biodegradability, making them suitable for various biomedical applications. Natural hydrogels can mimic the extracellular matrix, which offers a favorable environment for cell development and tissue regeneration. They are often used in wound healing, drug delivery, and tissue engineering applications. The choice between synthetic and natural polymers for hydrogel preparation depends on the desired mechanical properties, degradation behavior, biocompatibility, and specific application requirements. Synthetic and natural hydrogels have advantages and can be modified to meet the desired criteria for various biomedical and industrial applications [30].

Following the type of crosslink junctions, two basic categories of hydrogels are chemically and physically bonded together. Covalent bonds between different polymer chains create permanent junctions in chemically crosslinked hydrogels. This crosslinking type imparts excellent mechanical strength to the hydrogel [31]. However, it is essential to note that certain crosslinkers used in the chemical crosslinking process may have reported toxicity, requiring their extraction from the hydrogel before use to ensure safety. Physically crosslinked hydrogels, on the other hand, rely on non-covalent interactions, such as ionic contracts, hydrogen bonds, or hydrophobic interactions, to stop the dissolution of the polymer chains and keep the hydrogel structure stable [32]. Physically crosslinked hydrogels offer an alternative solution to address crosslinker toxicity concerns, as the interactions can be reversibly formed and disrupted, allowing for potential recycling or reprocessing of the hydrogel [33]. Both chemically and physically crosslinked hydrogels have several advantages and limitations, depending on the specific application requirements. The choice of crosslinking method depends on several factors, such as the desired mechanical properties, degradation behavior, biocompatibility, and the intended use of the hydrogel. Furthermore, research and development efforts in crosslinking techniques for hydrogels aim to enhance their mechanical properties, control degradation rates, improve biocompatibility, and explore innovative approaches that minimize the potential toxicity associated with crosslinkers [34].

### 3.2. Classification of Hydrogel Products

Various characteristics of hydrogels, such as their origin or source, composition, structure, crosslinking, network charge, durability, and reaction to external stimuli, are used to categorize them [36]. Some of the types are as follows:

#### 3.2.1. Based on Sources

##### Synthetic Source

Synthetic hydrogels are formed by polymerizing various synthetic monomers by ionic or covalent linking. These include the use of polyacrylates and glycolates monomers. Synthetic polymers, such as polyethene glycol, are water-soluble, biocompatible, and inert for medical applications. Polyethylene glycol (PEG) has linear and branched structures with two or more hydroxy groups, which further function with others to provide crosslinking in the hydrogel [37]. Polyvinyl alcohol (PVA) is a hydrophilic polyhydroxy polymer that is another example of a synthetic polymer that is widely used for creating hydrogels due to its versatile mechanical properties, adhesion, and elasticity [38]. Gulafshan et al. fabricated a PVA-alginate hydrogel for accelerated wound healing [39]. Other synthetically prepared hydrogels include poloxamers, poly(N-Isopropylacrylamide, and poly(propylene oxides) [40].

##### Natural Source

Natural hydrogels are formed using natural and hybrid polymers derived from plant, animal, and marine sources. Examples of natural polymers include chitosan, a natural mucoadhesive polysaccharide derivative of chitin obtained from the deacetylation of chitin; it is reportedly used to prepare hydrogels due to negligible toxicity and improved wound healing activity [41]. According to their results, Sheng et al. developed a novel photothermal chitosan incorporated hydrogel for improved wound healing [42]. Use of other natural polymers includes gelatin, collagen, cellulose, and starch, due to their biodegradation and lesser therapeutic toxicities [43].

#### 3.2.2. Based on Crosslinking

Crosslinked hydrogels are divided into chemical-mediated hydrogels and physical hydrogels based on the preparation method. Chemical hydrogels have more permanent linking than physical hydrogels [44].

##### Chemically Crosslinked Hydrogels

As permanent crosslinking is involved in this type of polymer, these are preferred over physical ones. Crosslinked agents are grafted onto the polymer backbone. It involves reactions such as polymer–polymer conjugation, enzyme-catalyzed reactions, and ionic reactions. The development of chemically crosslinked hydrogels with free radical polymerization between ß-cyclodextrin and favipiravir for sustained release delivery was reported [45]. The chemically crosslinked hydrogels formed with higher mechanical strength due to covalent bonding. Karoyo et al. reported the formation of a beta-cyclodextrin hydrogel by chemical crosslinking with hexamethyl diisocyanate [46].

##### Physically Crosslinked Hydrogels

These are self-assembled hydrogels produced by the interactions between molecules of hydrophobic groups, hydrogen bonds, and the electrostatic attraction of molecules [47]. Gabriela et al. fabricated porous chitosan-based hydrogels by physical crosslinking; NaOH was used as a crosslinking agent, and with the help of X-ray photoelectron spectroscopy, the physical properties were analyzed, and biocompatibility studies were tested [48].

#### 3.2.3. Classification Based on Response to Stimuli

Various external stimuli influence a hydrogel’s ability to expand and deflate. In reaction to specific physical or chemical stimuli, they experience an extent of collapse or shift in phase. Depending on how polymers respond to stimuli, hydrogels may be sensitive to changes in temperature, magnetic and electric fields, solvent composition, pH, sound, or molecular species [49].

## 4. Technologies Adopted in Hydrogel Preparation

Various technologies are employed to prepare hydrogels to control their composition, structure, and properties. These include crosslinking, polymerization, self-assembly, electrospinning, and microfluidics. Crosslinking involves chemically or physically linking polymer chains to enhance stability and mechanical strength. Polymerization techniques enable the synthesis of hydrogels from monomers, tailoring properties through reaction control. Self-assembly utilizes non-covalent interactions for spontaneous gel formation [50]. Electrospinning produces nanofibrous hydrogel scaffolds. Microfluidics manipulates fluids at the microscale for controlled hydrogel fabrication. Each technology offers unique advantages and is selected based on the hydrogel’s desired characteristics and intended applications [51].

One conventional method of hydrogel fabrication is the bulk polymerization method. The monomers and crosslinking agents are mixed in a solvent or aqueous solution [52]. The mixture is then subjected to a polymerization process, which can be initiated by heat, light, or chemical initiators. As the polymerization reaction progresses, the monomers crosslink to form a three-dimensional hydrogel network. The hydrogel is washed and purified to remove residual chemicals or unreacted monomers. This method is relatively simple and widely used for fabricating hydrogels with homogeneous structures.

### 4.1. Three-Dimensional Printing

Three-dimensional printing technology has become a powerful tool for fabricating complex structures, including hydrogels. The application of 3D printing in hydrogels offers numerous advantages and possibilities. Here are some critical aspects related to 3D printing for hydrogels:

***Customized and complex structures*:** Three-dimensional printing enables the precise fabrication of hydrogel structures with customized designs and complex geometries. This capability allows the creation of patient-specific implants, tissue scaffolds, or drug delivery systems tailored to individual needs [53].

***Layer-by-Layer fabrication*:** Three-dimensional hydrogel printing often utilizes layer-by-layer deposition techniques, such as extrusion-based techniques, inkjet, or stereolithography. These methods allow for the controlled deposition of hydrogel materials, facilitating the creation of intricate structures with high precision [54]. Qinghua Wu reported a process to fabricate micro-structured hydrogel scaffolds using 3D printing that produces chitosan hydrogel with highly flexible and ordered microfiber networks [55].

***Bio-ink development:*** Bio-inks are hydrogel-based materials formulated explicitly for 3D printing applications. These bio-inks possess suitable rheological properties to enable extrusion or deposition and can incorporate cells, growth factors, or other bioactive agents. Bio-ink development is an active area of research to enhance printability and ensure cell viability and functionality within the printed hydrogel structures [56].

***Tissue engineering and regenerative medicine*:** Three-dimensionally printed hydrogels significantly affect tissue engineering and regenerative medicine. By incorporating cells and bioactive molecules within the hydrogel matrix, 3D-printed constructs can mimic the architecture and functions of native tissues. This technology holds great promise for fabricating functional tissues and organs for transplantation or in vitro models for drug testing [57].

***Drug delivery systems*:** Three-dimensionally printed hydrogels can serve as platforms for controlled drug delivery. By incorporating drugs or therapeutic agents within the hydrogel matrix, 3D-printed constructs can release the payloads in a controlled manner, providing localized and sustained drug delivery for various applications, including wound healing and infection treatment.

***Bioprinting*:** Bioprinting refers to the precise deposition of cells, biomaterials, and bioactive factors to create complex 3D structures with cellular functionality. Bioprinting of hydrogels enables the fabrication of tissues with organized cellular patterns, creating possibilities for tissue engineering, regenerative medicine, and organ-on-a-chip technologies [58]. The applicability and printability of chitosan as a bioprinting solution were demonstrated in previous reports where investigators utilized 3D printers that can print 3D objects to implement the presence of cells within the ink [59,60].

Three-dimensional printing for hydrogels is continuously evolving, with ongoing research focused on improving printability, biocompatibility, mechanical properties, and scalability. It can potentially revolutionize the fabrication of hydrogel-based constructs for various biomedical applications. Hydrogels with intricate structures are developed using 3D printing, which is a versatile and beneficial manufacturing technology, even though these gels are frequently mechanically fragile. Three-dimensional printing has significant advantages over conventional processing techniques, including an easy operating process, accurate structural control, and cost effectiveness. Layer-by-layer printing allows for the creative and individual design of 3D-structured hydrogels for many practical purposes [61]. The three-dimensional printing of hydrogels involves different methods, including laser printing, extrusion printing, inkjet printing, etc. [62]. Wang et al. developed a 3D-printed antioxidant carboxy methyl cellulose (CMC)/e-polylysine hydrogel for skin wound repair; according to the study, hydrogels have excellent inhibitory effects against bacterial strains, accelerate tissue regeneration, and promote wound healing [63]. Miguel et al. developed a novel 3D-printed hydrogel for tissue regeneration using a polycaprolactone and silk sericin blend. There in vitro results show that 3D-constructed gels have good physical and mechanical properties with biocompatibility [64].

### 4.2. Electro-Spraying

The electro-spraying method is a versatile technique that has been widely employed for the fabrication of hydrogel-based materials. It uses an electric field to generate fine droplets or aerosols from a liquid polymer solution or dispersion. Here are some critical aspects related to the electro-spraying method for hydrogels:

***Process overview*:** Electro-spraying typically involves using a syringe pump to deliver the hydrogel solution or dispersion through a nozzle or capillary. An electric field is applied between the nozzle and a collector electrode, forming a Taylor cone at the nozzle tip. As the solution is ejected from the cone, it undergoes rapid solvent evaporation, forming solid hydrogel particles or fibers.

***Particle and fiber formation*:** The electro-spraying method allows for the fabrication of hydrogel particles with controlled sizes ranging from nanometers to micrometers. The size and morphology of the hydrogel particles can be tailored by adjusting the parameters, such as the polymer concentration, flow rate, applied voltage, and distance between the nozzle and the collector. The electro-spraying technique can also produce hydrogel fibers, which have applications in tissue engineering and drug delivery systems.

***Encapsulation of bioactive agents*:** Electro-spraying enables the encapsulation of bioactive agents, such as drugs, proteins, or growth factors, within the hydrogel particles. These bioactive agents can be incorporated into the hydrogel solution before electro-spraying, allowing for a controlled release over time. This capability makes electro-sprayed hydrogel particles promising for drug delivery applications.

***Control over material properties*:** The electro-spraying method controls the composition and properties of the hydrogel materials. Different hydrogel precursors, such as natural polymers (e.g., gelatin, alginate) or synthetic polymers (e.g., polyethylene glycol, polyvinyl alcohol), can achieve the desired material characteristics. Additionally, using crosslinking agents during the electro-spraying process can form crosslinked hydrogel particles or fibers, imparting stability and mechanical strength to the final product.

***Biomedical applications*:** Electro-sprayed hydrogel particles and fibers have applications in various biomedical fields. They can serve as carriers for drug delivery, tissue engineering scaffolds, wound dressings, or bioactive coatings on medical devices. The electro-sprayed hydrogel particles’ small size and high surface area facilitate their interactions with cells and tissues, making them suitable for targeted therapeutic delivery and tissue regeneration.

The electro-spraying method offers a flexible and efficient approach for fabricating hydrogel-based materials with controlled sizes and properties. Ongoing research should explore the potential of electro-sprayed hydrogels in various biomedical applications, with an aim to optimize their formulation and processing parameters for enhanced performance and functionality. A fragile liquid is formed during electro-spraying, and a bulk liquid jet splits into droplets with equal charges to aid in dispersion and prevent coagulation. Electrospinning (ES) uses a similar basic experimental setup as ES. It has a grounded substrate or collector for gathering the particles, a high-voltage power supply, a syringe pump, and a plastic or glass syringe with a metallic cap with a predefined diameter [65]. Zhao et al. fabricated a coaxial electrospray technique to produce microcapsules with hydrogel shells for encasing alginate and an aqueous liquid core in single-step tiny microcapsules [66]. Xiaqing et al. fabricated a PVA hydrogel through a single electro-spraying method for controlled drug delivery [67]. Era et al. fabricated a polyethylene glycol-based hydrogel by electro-spraying [68].

## 5. Chitosan as a Natural Bioactive Polymer

Chitosan is a versatile hydrophilic and cationic natural polysaccharide that is non-toxic, non-carcinogenic, and non-immunogenic. It comprises β-(1–4)-linked D-glucosamine and N-acetyl-D-glucosamine units. It is also known as soluble chitin and is made from chitin by alkaline deacetylation. Chitin comprises unbranched chains of β-(1–4)-2-acetamido-2-acetamido-2-deoxy-D-glucose. Chitin is found naturally in mushrooms, green algae, and some microorganisms, such as yeast and fungi. Also, it’s derived from invertebrates, such as crustacean shells or insect cuticles [69]. Chitosan’s physicochemical qualities, such as solubility, appearance, and rheological properties, are also influenced by its varying molecular weight and degree of acetylation [70]. Crosslinking, graft co-polymerization, carboxymethylation, etherification, and esterification must be highlighted as the primary methods to functionalize the chitosan structure among specific chemical processes to increase their capabilities.

The presence of amino and hydroxyl groups in chitosan allows for intriguing applications, as these can be changed to improve various aspects of this biopolymer [71,72,73,74]. The Food and Drug Administration (FDA) has classified this biopolymer as “Generally Recognized as Safe” (GRAS). Chitosan’s cationic nature causes it to form a range of forms in acidic conditions, including nano/microparticles, emulsions, fibers, hydrogels, films, and membranes [75]. In recent years, several ways for modifying the surface properties of chitosan have evolved. Several changes are chemical, while others are physical. Graft co-polymerization techniques have recently emerged to improve chitosan’s already exceptional characteristics. These approaches promise effective methods for engineering chitosan to include the desired features and functionalities [76]. Chitosan oligosaccharides (COS) are by-products of the enzymatic or chemical hydrolysis of chitin or chitosan; COS has a variety of unique biological properties, including anti-inflammatory, antibacterial, immunomodulation, and neuroprotection properties, as well as excellent water solubility and biodegradability with biocompatibility [77]. Chemical degradation, particularly acid hydrolysis, has been widely used in the mass manufacture of COS in the industry due to its ease of operation and low cost of manufacturing. Most commercially produced and used COSs are hetero-saccharides containing acetylated and deacetylated glucosamine units, such as chitosan [77].

Moreover, developing COS as a nanocarrier and scaffold material for tissue engineering has implications for biomedical applications. Additionally, it has been utilized to encapsulate other nanoparticles to enhance their mucoadhesive qualities [78]. Furthermore, chitosan and modified chitosan are frequently employed in tissue engineering research, particularly as composites for bone scaffolds. Chitosan is a polymer of choice for tissue engineering due to its solid structural resemblance to the natural glycosaminoglycan in the human extracellular matrix. In conjunction with drugs ranging from small particles to varied molecular weight peptides/proteins, chitosan nanoparticles, microparticles, injectable gels, films, and patches have been fabricated [70].

Regarding specific uses, chitosan is a polymer of great interest in biomedical research. It has been employed in tissue engineering, drug delivery, and skin wound healing research. Chitosan has been used commercially to treat obesity and hyperlipidemia as an alimentary supplement and hemostatic agent [75,79].

### 5.1. Effects and Mechanisms of Chitosan on Skin Wounds

Surgery, grazing, chronic ulcers, and other trauma can all result in wounds on the skin. Skin wound healing is a physiological process influenced by various factors, which can be promoted by good wound care and employs effective wound care materials [80]. Adaptable, stable, biodegradable, and widely applicable wound care materials should be able to keep wounds moist, stop bleeding, and take in waste [81]. Chitosan and its derivatives possess hemostatic, antibacterial, anti-inflammatory, film-forming, and analgesic properties, which may promote skin wound healing and prevent bacterial infection. Chitosan has antibacterial properties in wound dressings in four forms: hydrogel, fiber, sponge, and membrane [80]. Chitosan promotes wound healing by triggering inflammatory cells, macrophages, and fibroblasts, thereby enhancing the inflammatory phase. Chitosan and its derivatives will mostly play roles in the first three stages of skin wound healing.

### 5.2. Role of Chitosan in Stages of Skin Wound Healing

Tissue regeneration is a complex process involving multiple molecular mechanisms. One key mechanism is the activation of specific signaling pathways that regulate cell proliferation, migration, and differentiation [82]. When a wound occurs, dermal cells, such as fibroblasts and keratinocytes, play essential roles in healing. Fibroblasts produce the extracellular matrix (ECM), which provides structural support and guides cell migration. They also secrete growth factors and cytokines that stimulate cell proliferation and tissue remodeling [83]. Keratinocytes, the predominant cell type in the epidermis, contribute to wound healing by migrating to cover the wound bed, forming a new epithelial layer [84]. These cells also produce various growth factors and cytokines that promote cell proliferation and angiogenesis. In the initial inflammatory phase of wound healing, immune cells release cytokines that recruit fibroblasts and initiate ECM synthesis. Fibroblasts then migrate to the wound site and proliferate, depositing collagen and other ECM components [85]. This provisional matrix is a scaffold for cell migration and guides tissue regeneration. The proliferative phase involves the migration and proliferation of keratinocytes to resurface the wound bed.

Additionally, new blood vessels, a process called angiogenesis, form to provide oxygen and nutrients to the healing tissue [86]. In the final remodeling phase, excessive ECM is broken down, and the wound undergoes structural reorganization. Collagen fibers become more organized, and the wound contracts, reducing its size [87]. Overall, tissue regeneration relies on a complex interplay of cellular and molecular events, including cell migration, proliferation, ECM synthesis, and remodeling. Dermal cells, such as fibroblasts and keratinocytes, contribute significantly to these processes and play crucial roles in the healing of wounds.

The stages are as follows: The first stage is “coagulation and hemostasis”, where chitosan and its derivatives have a more significant hemostatic impact and are unrelated to the conventional coagulation system, i.e., the activation of coagulation factors as well as helping in the prevention of bleeding by enhancing platelet and erythrocyte aggregation, increasing leukocytes, and blocking fibrinolysis. Chitosan will help inflammatory cells such as macrophages eliminate bacteria and necrotic tissue from the wound during the “inflammation” stage. The inflammatory phase ends and the proliferative phase of wound healing begins promptly. Chitosan may promote fibroblast proliferation by forming polyelectrolyte complexes with growth factors, heparin, cytokines, or other proteins at the wound site. The final stage, remodeling, occurs to complete the skin repair process [81,88]. All living things, including people, can regenerate. This is accomplished by molecular mechanisms controlled by the gene expression program that governs repair, growth, and renewal [89].

**Figure 3:**
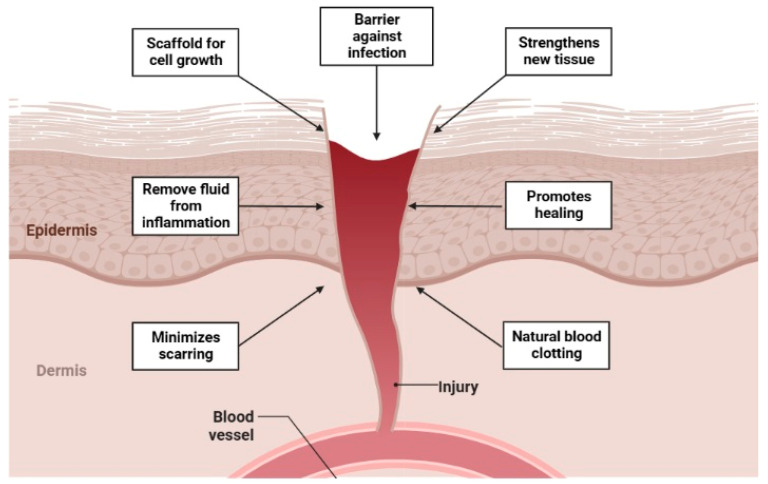
Mechanism of action of hydrogels in skin infections.

Skin epithelial cells are labile elements that are constantly removed in the stratum corneum by the keratinocyte desquamation process and replaced in the basal layer by differentiated elements derived from stem cell proliferation and differentiation. Cell renewal is affected by various circumstances, including trauma, hormonal impacts, skin diseases, and individual well-being. However, in the case of a wound lesion, the cutaneous regenerating process is inversely related to the progression of the infection [90]. Healing a skin wound demonstrates an exceptional cellular function process that is unique. Cell development, differentiation, and tissue morphology are all part of the regeneration process. The coagulation cascade and inflammatory pathways are also involved at the cellular level. Several cells are involved, including fibroblasts, keratinocytes, endothelial cells, neutrophils, monocytes, macrophages, and lymphocytes [91]. Regeneration involves forming billions of cells and using a highly evolved feedback loop to reject damaged or unfit cells. Active processes are terminated by silencing genes while the regeneration process progresses. The tissue regenerative process is one of the most complex biological phenomena, including a wide range of cell types, growth factors, cytokines, and metabolites [91]. Due to this complication, the wound healing process is separated into three stages: inflammatory, proliferation, and remodeling [92]. The stages are as follows: The first stage is “coagulation and hemostasis”, where chitosan and its derivatives have a more significant hemostatic impact and are unrelated to the conventional coagulation system—i.e., the activation of coagulation factors—by helping in the prevention of bleeding by enhancing platelet and erythrocyte aggregation, increasing leukocytes, and blocking fibrinolysis (Figure 3). Chitosan will help inflammatory cells such as macrophages eliminate bacteria and necrotic tissue from the wound during the “inflammation” stage. The inflammatory phase ends and the proliferative wound healing begins promptly [81,88]. Shi et al. studied these phenomena, wherein a chitosan/silk hydrogel sponge was prepared through a freeze-drying process. This sponge exhibited exceptional physical and structural characteristics, which made it suitable for wound healing and can be used as a scaffold for exosomes. Combining exosomes and hydrogels might effectively increase skin wound healing in a STZ-induced diabetic rat model by increasing re-epithelialization, ECM deposition, and remodeling, as well as by enhancing angiogenesis and neuronal growth [93]. A chitosan–alginate complex hydrogel was developed by Wei et al.; the hydrogels demonstrated appropriate rheological properties and pore size, which made cell adhesion possible. The implementation of hydrogels could offer a three-dimensional environment for cell cultures, which can improve both cell–cell and cell–ECM interactions. Through the incorporation of FGF and VE-cadherin, the PI3K/AKT signaling pathways may be utilized to improve cell proliferation and migration. Furthermore, a histological analysis revealed that a full-thickness skin defective model had a better wound-healing effect [94]. Moreover, a recent review indicated improved effects of chitosan as polymeric carrier materials in accelerating the wound repair and healing process suggesting the efficacy in skin-tissue engineering [95].

### 5.3. Antibacterial Activity of Chitosan in Skin Wound Healing

The moist, nutrient-rich environment of the wound provides perfect conditions for bacterial development. Bacterial infections develop when the host immune system cannot eliminate all invading germs. As a result, the antibacterial qualities of skin wound dressings must be carefully evaluated. Due to its high antibacterial characteristics, chitosan is commonly employed in wound therapy. Disrupting bacterial cell walls and membranes, chelating small amounts of metallic cations, engaging with internal targets, and depositing on bacteria are currently acknowledged possible mechanisms of chitosan for antibacterial activity [96]. Abid et al. employed electro-spun chitosan nanofibrous mats that demonstrated remarkable antibacterial potential by targeting the negatively charged bacterial cell wall by rupturing the cell membrane, increasing cell perforations, and resulting in intracellular protein and nucleotide constituent leakage [97]. Cai et al. prepared electro-spun non-fibrous chitosan and silk fibroin mats that demonstrated exceptional antibacterial and wound healing potential [98]. Zhao et al. used electrospinning to create sericin–chitosan nanofibers, and the produced membranes had great potential for wound healing applications. Chitosan/sericin nanofibers have shown promising antibacterial efficacy against Gram-negative and Gram-positive bacteria, in addition to their consistent fiber morphology, high biocompatibility, and cell proliferation capabilities [98]. The antibacterial effect of chitosan relies on its polycationic structure. Electrostatic interactions between the polycationic structure of chitosan and its derivatives and the predominantly anionic components of the microorganisms’ surface, such as Gram-negative lipopolysaccharide and cell surface proteins, is crucial for antibacterial activity—as the pH of the environment is lower than the pKa of these substances [99]. The antibacterial activity process varies between Gram-positive and Gram-negative bacteria due to differences in the features of their cell surfaces. Several studies have shown that the antibacterial activity of Gram-negative bacteria is better than that of Gram-positive bacteria [100]. In a different study, it was determined that Gram-positive bacteria are more susceptible, potentially due to the barrier presented by the Gram-negative outer membrane [101,102]. It is being suggested that alterations in the electronic negativity of the cell surface fluctuate according to the growth phase, potentially resulting in variations in cell susceptibility to chitosan [103]. Examples of the antibacterial activity of chitosan-based hydrogels are shown in Table 1.

## 6. Preparation, Properties, and Process Optimization of Hydrogel-Based Grafted Chitosan

### 6.1. Preparation of Chitosan Crosslinking

**Figure 4:**
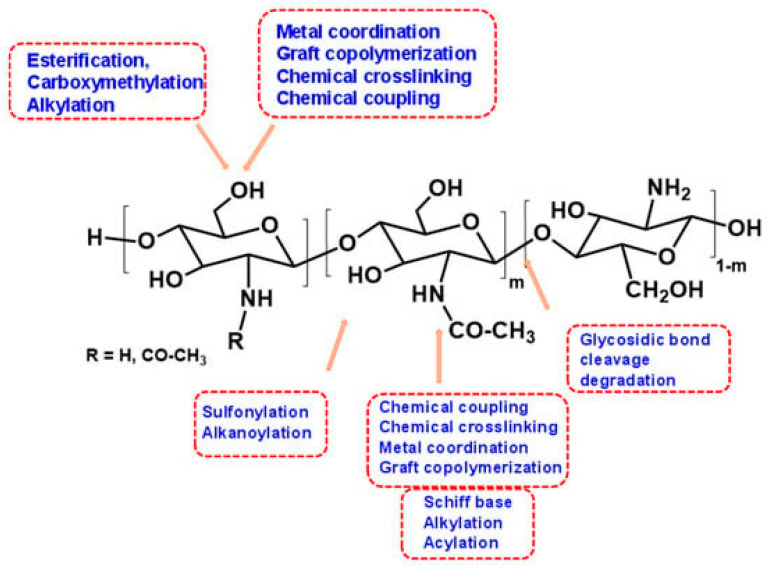
Functional groups in chitosan polymer that can be chemically modified. Reproduced with permission under CC BY 4.0 license [109].

Intermolecular hydrogen and hydrophobic and ionic interactions hold the polysaccharide chains of chitosan together. The ionic strength and molecular weight have an impact on these interactions. The different functional groups in chitosan polymers that can be converted into hydrogels by different mechanisms are shown in Figure 4. The crosslinking of chitosan polymers is required to enhance chitosan features for drug delivery, such as stability and durability. Depending on how the chitosan is prepared and crosslinked, different types of chitosan-based hydrogel networks exist. Chemically crosslinked hydrogels are formed by covalent linking, which is a four-stage process involving the crosslinking of structures, the formation of the hybrid polymer network, an interpenetrating network, and a semi-interpenetrating network [107,108].

### 6.2. Properties

It has been demonstrated that chitosan inhibits the growth of yeast strains, filamentous fungi, and bacteria. Chitosan has also been identified as an antibacterial agent; however, it is unclear whether it has this ability, given that its nature has been attributed to several distinct processes. When used against Gram-positive and Gram-negative bacteria, chitosan exhibits a wide range of effects and a high mortality rate. While most Gram-negative microorganisms are not hazardous to mammals, the size of a substance’s molecular structure is thought to affect how bactericidal it is [109].

### 6.3. Process Optimization

It is necessary to optimize the preparation methods to achieve the desired hydrogel structure and properties for progressive wound healing. The crosslinker and monomer concentration amount affects the hydrogel’s swelling and mechanical strength. Kho rasani et al. optimized the process parameters of polyvinyl alcohol chitosan and nano zinc oxide hydrogels for wound healing by applying response surface methodology [110]. Poonam et al. prepared phospholipid microemulsion-based hydrogels for the topical delivery of lidocaine and prilocaine. According to their results, the optimized batch shows good histopathology and enhanced drug permeation [111]. Chintan et al. developed % fluorouracil-loaded thermosensitive hydrogels and evaluated the effects by identifying the quality target product profile and critical quality attributes and checking their effect on the optimized formulation [112].

### 6.4. Self-Healing Chitosan Hydrogels

Recent research identified self-healing (SH) hydrogels as a novel class of soft matter with the remarkable capacity to regain structure and function following the application of an external stimulus, such as a needle injection. To fill a hollow cavity and develop a coating on a surface or promote wound healing, the SH hydrogels are valuable materials that can be easily administered by injections at a specific spot on the body. Additionally, by adding bioactive substances, they provide effective drug delivery devices for use in tumours or on wounds for localized action [113]. To create SH hydrogels, non-covalent or reversible covalent bonds that rupture under external tension and re-establish when the stress has been removed are used. As a result, the SH mechanism, which enables the hydrogel to repair damage and assume new shapes, is based on the dynamic equilibrium between the dissociation and recombination of numerous contacts. Imine, boronate, oxime, disulfide, and arylhydrazone bonds, or Diels-Alder processes, are frequently utilized reversible dynamic bonds to construct SH hydrogels, whereas non-covalent contacts include hydrogen bonds, or ionic, host–guest, and hydrophobic interactions [114]. The main drawback of polymers such as polyvinyl alcohol, poly(ethylene oxide), poly(N-isopropyl acrylamide), polyacrylic acid, poly stearyl methacrylate, poly (N, N-dimethyl acrylamide), or polyoxymethylene acrylate is that they are non-biodegradable, which restricts their use in vivo. To overcome these problems, using natural polymers with biodegradable properties was acceptable. Chitosan is a naturally originating polymer with a biodegradable nature, which was broadly used by researchers in the medicinal field [115]. Guo et al. fabricated injectable quaternary ammonium chitosan and tannic acid hydrogels. This hydrogel system is constituted by crosslinked ionic and hydrogen bonds between quaternary ammonium salts and tannic acid with self-healing properties. According to their findings, the given hydrogel possesses a higher super oxide scavenging activity, broad-spectrum antibacterial activity, and hemostatic capability. In vivo, the results show a good clotting time and accelerated wound healing [116].

**Figure 5:**
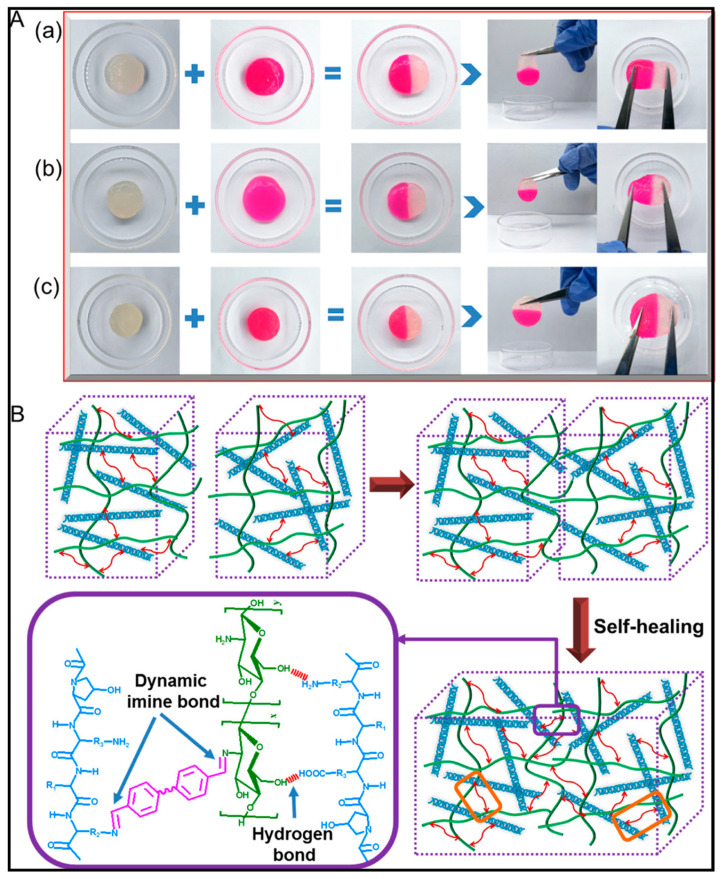
Illustration of Cut–Heal properties of the hydrogel obtained from collagen–chitosan at a ratio of (**a**) 1/1, (**b**) 1/2, and (**c**) chitosan alone (**A**). Systemic representation of self-healing mechanisms showing hydrogen bond and imine bond formation (**B**). Reproduced with permission from [119] under Copyright 2020, American Chemical Society.

A reported study by Zhang et al. developed a carboxymethylated chitosan hydrogel with mild photothermal stimulation for wound healing via Schiff bonds. After covalently incorporating GO-BPEI (branched polyethyleneimine grafting graphene oxide), better mechanical strength and photothermal conversion properties of a GO-BPEI/carboxymethylated chitosan/aldehyde-terminated polyethylene glycol hydrogel have been witnessed. In vivo, studies and histopathological results reveal accelerated wound healing by improving collagen fibers [117]. The use of traditional herbal medicines to formulate new and efficacious drug delivery systems has gained importance; Zeng et al. have introduced chitosan puerarin, which is an injectable and self-healing hydrogel for diabetic wound healing. According to their study, it inhibits miR-29a- and miR-29b1-mediated inflammation in mice skin [118]. The demonstration of the Cut–Heal properties of the hydrogel obtained from collagen–chitosan is shown in Figure 5.

## 7. Chitosan-Based Nanoparticle-Incorporated Hydrogels

Nanotechnology has enabled fresh approaches to overcoming obstacles in various fields during the past few decades. In the biomedical sciences, where nanotechnology platforms have enormous potential for improving how we diagnose, prevent, and treat human disease, the capacity to customize nanomaterials that can interact with biological systems in different ways is particularly appealing. A new form of nanomaterial known as nanoparticle–hydrogel superstructures is comprised of hydrated, crosslinked polymeric networks combined with nanoparticles. In recent years, both hydrogel and nanoparticle systems have seen significant advancements [120]. In addition to customizing and enhancing hydrogels’ mechanical and aesthetic properties, the integration of nanoparticles has been explored for application in hydrogels. For instance, stimuli-sensitive hydrogels loaded with enzymes or nanoparticles containing drugs have been developed for drug delivery applications; for example, magnetic nanoparticles can make hydrogel particles magnetic, facilitating separation and recycling processes, and hydrogels that contain nanoparticles can be used to study their swelling properties [121].

Shafique et al. designed a hydrogel based on hyaluronic acid, pullulan, and polyvinyl alcohol and loaded it with chitosan-based cefepime nanoparticles for potential use in cutaneous wound healing. The given hydrogel was evaluated against dynamic light scattering and other analytical characterizations. According to their results, it shows novel crosslinking, thermal stability, a good swelling index, and improved wound healing according to animal studies [122]. As metallic nanoparticles are widely used for their antimicrobial activity, metallic-nanoparticle-fabricated hydrogels come into the picture. Some of the reported studies are as follows. Mohmed et al. synthesized carboxymethyl chitosan silver nanoparticle hydrogels and evaluated them against bacterial strains such as *Staphylococcus aureus*, *Bacillus subtilis*, and *Streptococcus faecalis*, showing good results [123]. Zhang et al. fabricated a sodium alginate chitosan oligosaccharide zinc oxide nanoparticle-based hydrogel for accelerated wound healing. According to their results, it provides a moist and antibacterial environment for wound healing, with desirable porosity and swelling with better biocompatibility [124]. George et al. developed a novel hydrogel containing Phyto-derived quercetin isolated from onion peel waste and dialdehyde cellulose from sugarcane bagasse, which were synthesized with zinc oxide nanoparticles embedded in the crosslinked structure. Different analytical methods characterized it. The biocompatibility and anticancer properties of the QE/OPD-loaded nanohybrid hydrogels were established against normal L929 murine fibroblast cells and A431 human skin carcinoma cell lines, respectively [125].

### 7.1. Chitosan/Drug Hydrogels

Chitosan has received much attention as a potential raw material for hydrogels because of the polymer’s biocompatibility, biodegradability, and low toxicity. Creating intelligent chitosan hydrogels using biomimetic principles has generated much research interest in materials science and engineering [126]. Physical and chemical chitosan (CS) hydrogels are well known for being highly effective and practical systems that can prolong the release of hydrophilic, hydrophobic, or even macromolecular drugs [127]. Hoang et al. developed a double crosslinked hydrogel from chitosan oligosaccharide/alginate using Norborne-tetrazine click reaction for ketoprofen delivery [128]. Shah et al. developed a solvent casting that was used to create injectable hydrogels made of chitosan and CMC-g-PF127. The injectable hydrogels for curcumin have controlled release characteristics, viscoelastic behavior, and good swelling qualities [129].

**Figure 6:**
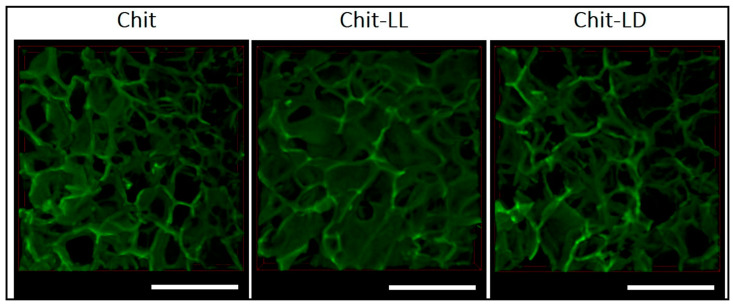
CLSM images of the 3D macroporous hydrogel samples produced from chitosan (Chit) and two co-polymers: chitosan-g-oligo (L, L-lactide) (Chit-LL) and chitosan-g-oligo (L, D-lactide) (Chit-LD). Reproduced with permission under CC BY 4.0 license [130].

In one of the studies, chitosan-g-oligo (L, L-/L, D-lactide) co-polymers were used to create novel matrices (Figure 6). Two matrices were fabricated: 2D films through solvent casting and 3D macroporous hydrogels through lyophilization of co-polymer solutions [130]. The co-polymers were synthesized through solid-state mechanochemical synthesis by combining chitosan with semi-crystalline oligo (L, L-lactide) (Chit-LL) or amorphous oligo (L, D-lactide) (Chit-LD).

### 7.2. Chitosan/Bioactive Substance Hydrogels

Bioactive materials/substances can influence their surroundings by inducing a biological reaction. Bioactive materials based on metals, polymers, ceramics, and their composites play an essential role in tissue engineering domains, such as bone, neuron, muscle, and skin regeneration [131]. Several chitosan and chitin derivatives and their composites, including a wide range of functional bioactive materials, have been researched for skin wound healing and tissue engineering to introduce desirable qualities. Sofia et al. employed a mixture of chitosan and gelatin to develop 3D printable hydrogels, which were further characterized for physiochemical and biological properties. It also showed self-healing features and proof of biocompatibility. This opened the way for gelatin–chitosan hydrogels as a biomaterial, which can be 3D printed and used in tissue engineering [132]. Mostafa et al. fabricated a novel hydrogel made from sodium alginate and chitosan with aloe vera extract and honey that might be applied to treat and heal wounds. It was characterized by physical properties such as swelling, porosity, density, and mass loss. Also, biocompatibility was checked by analyzing cell viability and hemolytic activity. Based on the results of this study, alginate and chitosan led to the formation of a biocompatible structure that can be used as a medium to repair tissue and wounds [133]. Kun-Chih Cheng et al. prepared a novel chitosan–cellulose nanofiber composite that is a self-healing hydrogel with adjustable self-healing properties. The self-healing properties, ability to inject, and other rheological properties of the hydrogel were also analyzed for cell proliferation and gene expression [134].

### 7.3. Chitosan-Reduced/Capped Metallic Particle Gels

**Figure 7:**
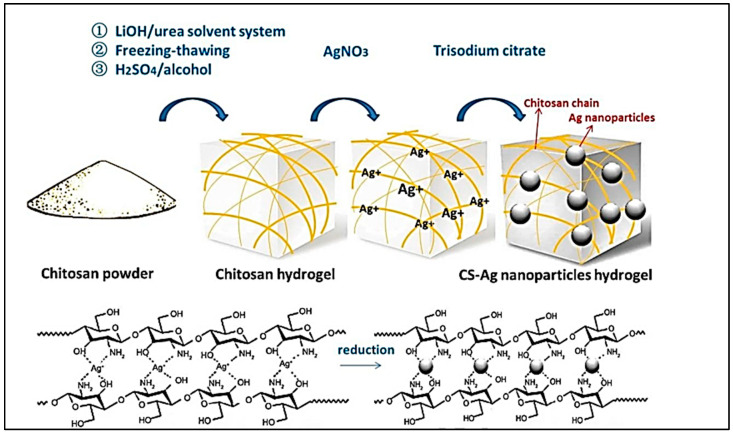
The synthesis of Ag nanoparticles in the chitosan hydrogel network is depicted schematically (adapted and reproduced with permission from [137]).

Dana et al. synthesized stable chitosan–PVA-based hydrogels through a double crosslinking procedure, and, later, its antimicrobial activity was enhanced via the in-situ generation of silver nanoparticles in hydrogel under UV irradiation. The cytotoxicity and in-vitro antibacterial activity of the hydrogels were investigated. The study showed that the swelling property, mechanical property, and antimicrobial activity recommended their use in antibacterial devices with other biomedical applications [102]. Fernanda et al. prepared chitosan-coated gold nanoparticle hydrogels by crosslinking the chitosan to eliminate the use of toxic crosslinkers. The resultant hydrogel showed outstanding properties when compared with chitosan hydrogels crosslinked by glutaraldehyde. The hydrogel did not show cytotoxicity, and it enhanced the properties that are suitable for bioapplication [135]. Yajuan et al. fabricated a chitosan hydrogel that is reinforced by silver nanoparticles to increase the mechanical and antibacterial properties for accelerating wound healing. The hydrogels remained structurally intact after applying strain and showed excellent antibacterial performance [136]. Investigations demonstrated that if the hydrogel is synthesized as a biomaterial with applications in skin (or oral) dressing, the retention capacity and retention kinetics in water and simulated physiological situations must be evaluated. The hydrogels that were developed in this study are characterized by their small size and porous structures. Thus, it results in swelling rates that are relatively high. The in-situ synthesis of silver nanoparticles under UV irradiation enhanced the inherent antibacterial activity of chitosan [102]. In addition, crosslinking with AuNP30 enhanced the biocompatibility of the hydrogel. The crosslinking of chitosan hydrogel by AuNP30 did not exhibit cytotoxicity against MEF cells [135]. The schematic presentation of the synthesis of Ag nanoparticles in the chitosan hydrogel network is depicted in Figure 7.

### 7.4. Other Chitosan Composite Hydrogels

**Figure 8:**
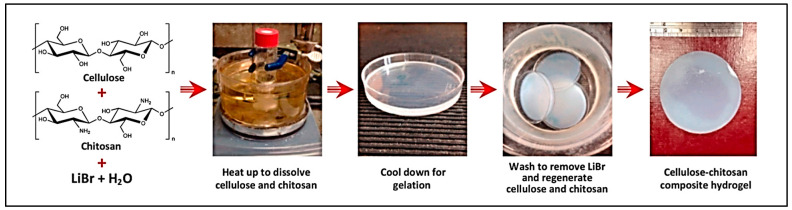
Fabrication of cellulose–chitosan composite hydrogel in concentrated LiBr solution (adapted with permission from [139]).

Yiwen et al. prepared a novel lignin–chitosan–PVA composite hydrogel for wound dressing in which the incorporation of lignin led to increased mechanical properties as a wound dressing. The prepared composite hydrogel was evaluated in a murine wound model and showed significant wound healing. This provided a new opportunity for efficient skin wound care [138]. Yang et al. fabricated a mesoporous cellulose–chitosan composite hydrogel that is bio-sorbent of heavy metals. It was seen that the mechanical property of the mesoporous cellulose–chitosan composite hydrogel was much more than that of a cellulose–chitosan hydrogel. This study demonstrated an innovative method to fabricate bio-sorbents from abundant and renewable polymers (Figure 8) [139].

### 7.5. Evaluations of Chitosan-Based Hydrogels

The generalized evaluations of chitosan-based hydrogels involve assessing various critical aspects of the hydrogel, including its physical, chemical, mechanical, and biological properties. Some standard evaluation methods for chitosan-based hydrogels include:

***Morphology analysis*:** The morphology of the hydrogel can be examined using techniques, such as scanning electron microscopy or transmission electron microscopy, to observe the internal structure, pore size, and distribution of the hydrogel network [140].

***Swelling behavior*:** The swelling behavior of the hydrogel can be evaluated by measuring the equilibrium swelling ratio or water uptake capacity [141]. This provides insights into the hydrogel’s ability to absorb and retain water, which is essential for drug delivery and wound healing applications.

***Mechanical properties*:** The mechanical properties of chitosan-based hydrogels, such as their elasticity, compressibility, and tensile strength, can be determined through mechanical testing methods, such as compression testing or tensile testing. These evaluations provide information about the hydrogel’s structural integrity and suitability for specific applications [142].

**Figure 9:**
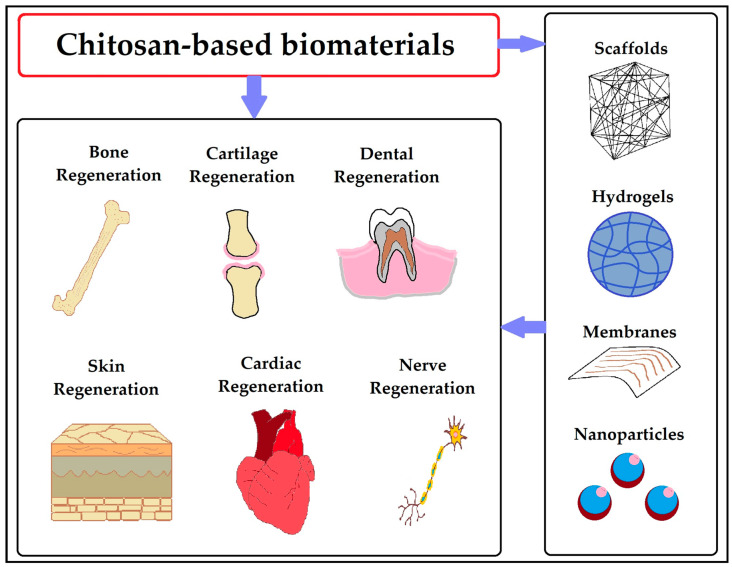
Chitosan-based biomaterials for tissue regeneration. Reproduced with permission under CC BY 4.0 license [144].

***Degradation studies*:** The degradation behavior of chitosan-based hydrogels can be investigated by monitoring the mass loss or changes in molecular weight over time. Degradation studies help assess the hydrogel’s stability and degradation rate, which are essential considerations for applications requiring drug-controlled release or tissue regeneration [143]. The various applications of chitosan biomaterials for tissue regeneration are shown in Figure 9.

***Biocompatibility assessment*:** The biocompatibility of chitosan-based hydrogels is evaluated through in vitro and in vivo studies. Cell viability assays, such as MTT assays or live/dead staining, can determine the cytotoxicity and cell compatibility of hydrogels. Animal studies can also evaluate the hydrogel’s host response and tissue compatibility [145].

***Drug release kinetics*:** For chitosan-based hydrogels used in drug delivery, the release kinetics of incorporated drugs can be evaluated [146]. This involves monitoring the release profile of the drug over time using techniques such as UV-visible spectroscopy or high-performance liquid chromatogram. These evaluations provide valuable information about the performance and suitability of chitosan-based hydrogels for various applications, guiding their optimization and development. It is important to note that specific evaluation methods may vary depending on the intended application and research objectives.

## 8. Chitosan Hydrogels Are Currently in Clinical Trials, and Patents

Chitosan hydrogels have emerged as a promising biomaterial in recent years, leading to their investigation in clinical trials for various medical applications. These trials have evaluated chitosan hydrogels’ safety, efficacy, and feasibility in diverse areas, including regenerative medicine and drug delivery. In regenerative medicine, chitosan hydrogels have great potential as scaffolds for tissue engineering and wound healing [147]. The unique properties of chitosan, such as biocompatibility, biodegradability, and antimicrobial activity, make it an attractive material for promoting tissue regeneration and facilitating the healing process. Clinical trials continue to assess chitosan hydrogels’ effectiveness in promoting the repair and regeneration of damaged tissues, such as cartilage, bone, skin, and nerve tissues. Chitosan hydrogels are also being investigated in clinical trials for drug delivery applications [148]. The versatile nature of chitosan allows the incorporation of various therapeutic agents, such as drugs, growth factors, and genes, within the hydrogel matrix. This enables the controlled and sustained release of the therapeutic payload, enhancing treatment outcomes and reducing potential side effects. Clinical trials are exploring the potential of chitosan hydrogels as local drug delivery systems for conditions such as cancer, chronic wounds, and ocular diseases [149]. Through these ongoing clinical trials, researchers aim to gather valuable data on the safety, efficacy, and optimal usage of chitosan hydrogels in real-world medical settings. The results obtained from these trials will provide insights into the potential of chitosan hydrogels as innovative solutions for addressing unmet medical needs, paving the way for their future clinical applications and commercialization. Several concerns with chitosan-based hydrogels must be addressed. Chitosan, for example, has a low solubility, and hydrogels have poor mechanical properties, limiting their usage in medical devices [150]. Polysaccharides have been employed in wound healing dressings for tissue regeneration due to their low toxicity and positive compatibility profile. However, due to the lack of protein structure, natural polysaccharides have very poor biostability and difficulties creating a “matrix” to cover the damaged tissue during wound healing, encouraging wound contraction and scar formation [151]. Furthermore, the development of temperature- or pH-responsive and functionalized biomaterials, such as chitosan-based hydrogels, necessitate the use of toxic chemicals, which are both expensive and dangerous (Table 2 and Table 3).

## 9. Challenges with Chitosan Hydrogels

Chitosan hydrogels have great potential in various biomedical applications, including wound healing, drug delivery, and tissue engineering. However, several challenges need to be addressed to exploit their benefits entirely. Here are some key challenges associated with chitosan hydrogels:

***Mechanical strength*:** Chitosan hydrogels often exhibit weak mechanical properties, limiting their use in load-bearing applications [161]. The inherent brittleness and low elasticity of chitosan can lead to poor hydrogel stability and structural integrity, particularly under mechanical stress. Enhancing the mechanical strength of chitosan hydrogels is essential to ensure their durability and suitability for demanding applications.

***Swelling behavior*:** Chitosan hydrogels tend to absorb a significant amount of water, resulting in excessive swelling. This swelling behavior can cause dimensional instability, loss of mechanical properties, and hinder the controlled release of encapsulated drugs or bioactive molecules [107]. Balancing the swelling properties of chitosan hydrogels to maintain structural integrity while allowing sufficient water uptake is crucial for their optimal performance [162]. A reported study indicates that pH change is a crucial factor in controlling the swelling properties of chitosan. Due to its hydrophilic nature and polyelectrolyte polysaccharide properties, chitosan undergoes swelling in aqueous environments. The change of pH is facilitated by the modification of -NH2 groups. The hydrophobic repulsion is caused by the protonation of the -NH2 group at acidic pH. Crosslinking of chitosan molecule in hydrogels leads to rigidity, consequently affecting the pH-dependent swelling. The swelling of the hydrogel depends on pH, as until the pH reaches 3, no swelling occurs and an increase in pH leads to an increase in swelling [163].

***Gelation kinetics*:** The gelation process of chitosan hydrogels can be relatively slow, leading to prolonged gelation times. This can limit their clinical utility, especially when rapid gel formation is required, such as for minimally invasive applications or on-site wound treatment [164]. Developing strategies to improve the gelation kinetics of chitosan hydrogels is necessary for their practical implementation.

***Contamination and sterility*:** Chitosan, derived from natural sources, can contain impurities, such as endotoxins and allergenic substances. Ensuring the purity and sterility of chitosan and its derivatives is crucial to minimize potential adverse effects and promote safe clinical use [165]. Rigorous purification and sterilization techniques must be implemented during the fabrication process to eliminate contaminants and maintain the biocompatibility of chitosan hydrogels.

***Biodegradation control*:** Chitosan hydrogels can undergo rapid enzymatic degradation in the presence of chitosanases and lysozymes, limiting their long-term stability and durability [166]. Controlling the biodegradation rate is essential to match the intended application, allowing sufficient time for tissue regeneration or drug release while maintaining the structural integrity of the hydrogel. Strategies such as chemical crosslinking or incorporating degradation-controlling agents can be explored to achieve the desired degradation profile.

***Scalability and manufacturing*:** Scaling up the production of chitosan hydrogels while maintaining consistent quality can be challenging [167]. The synthesis and fabrication processes of chitosan hydrogels need to be scalable, cost-effective, and reproducible to meet the demand for clinical translation. Developing robust manufacturing techniques and optimizing process parameters are necessary for the large-scale production of chitosan hydrogels [168]. Addressing these challenges through ongoing research and development efforts will contribute to overcoming the limitations of chitosan hydrogels and unlocking their full potential for various biomedical applications. By improving their mechanical properties, swelling behavior, gelation kinetics, purity, biodegradation control, and manufacturing scalability, chitosan hydrogels can be optimized for enhanced performance, safety, and clinical efficacy.

## 10. Conclusions

In conclusion, chitosan hydrogels offer great promise in wound healing due to their unique properties and favorable outcomes in this case study. Continued research and development of chitosan-based hydrogels will contribute to advancing the field of wound healing and provide effective solutions for improving patient care. Chitosan-based hydrogels have demonstrated promising results in the management of dermal infections. Their antimicrobial properties, biocompatibility, and wound-healing properties make them valuable therapeutic options. However, further research is necessary to optimize the formulation, investigate the long-term effects, and conduct clinical trials to establish their safety and efficacy in real-world settings. Chitosan-based hydrogels hold great potential for addressing the challenges associated with dermal infections and may pave the way for novel treatment modalities in dermatology.

## Figures and Tables

**Table 1:** Different types of chitosan-based hydrogels for antibacterial activity.

Bacterial Species	Hydrogel Type	Ref.
*E. coli* and *S. aureus*	Polyvinyl alcohol (PVA)/N–succinyl chitosan (NSCS)/lincomycin hydrogels	[104]
*E. coli*	PEG–Chitosan Hydrogel	[100]
*E. coli* and *S. aureus*	Chitosan/Alginate Hydrogel Dressing Loaded FGF/VE-Cadherin	[94]
*E. coli, S. aureus* and *P. aeruginosa*	PVA/Starch/Chitosan Hydrogel Membranes with Nano Zinc oxide	[105]
*S. aureus*	Chitosan/PVA-Based Hydrogel Films	[106]

**Table 2:** Summarized patented chitosan-based hydrogels used in biomedical applications and skin wound healing.

Patent Title	Application	Patent No.	Year	Ref.
Dextran-chitosan-based in-situ gelling hydrogels for biomedical applications	Tissue engineering applications to prevent tissue ingrowth	US20110076332A1	2010	[152]
Skin repair composition containing chitosan hydrogel	Repairing/healing chronic wounds or acute wound-type skin damage	JP2005517043A	2002	[153]
Composite chitosan hydrogel dressing, as well as preparation method and applications thereof	Extended drug delivery and wound healing	CN104707164B	2015	[154]
Chitosan-containing hydrogel and method of cosmetic skin care with its use	Cosmetic skin care	RU2667130C1	2017	[155]
Pseudo-thermosetting neutralized chitosan composition forming a hydrogel and a process for producing the same	Drug delivery system	US8507563B2	2004	[156]
Enhanced targeted drug delivery system via chitosan hydrogel and chlorotoxin	Drug delivery to tumour cells, such as glioma	US9522114B1	2015	[157]
Chitosan hydrogel derivatives as a coating agent with a broad spectrum of antimicrobial activities	Antimicrobial water shield coating agent.	US9750847B2	2009	[158]
Cross-linkable chitosan composition for producing a chitosan hydrogel	Used in pharmaceuticals, in the food industry, as a glue, as a lubricant or as a drilling servicing fluid	NZ584996A	2008	[159]

**Table 3:** Examples of chitosan-based hydrogels in clinical trials/marketed products.

Hydrogels	Application	Ref.
Tegasorb ^®^ 3M	Chitosan particles swell when they absorb exudate, generating a soft gel. Leg ulcers, sacral wounds, and chronic wounds can all benefit from this treatment.	[25]
Chitoseal ^®^ Abbott	It has excellent biocompatibility and hemostatic properties. Helpful for bleeding wounds	[160]
Chitoflex ^®^ HemCon	Antibacterial and biocompatible. For stuffing into a wound track to control severe bleeding	[25]
Chitopoly ^®^ Fujispinning	Antimicrobial clothing made with chitosan and polynosic Junlon poly(acrylate).	[25]
Chitopack C ^®^ Eisai	Repairs damaged bodily tissues and regenerated skin regularly.	[160]
Chitoderm ^®^ plus	Adsorbent properties.	[160]

## Abbreviations

3D—Three dimensional; SH—Self healing; PEG—polyethene glycol; PVA—Polyvinyl alcohol; COS—Chitosan oligosaccharides.
